# Effect of menopause on salivary parameters and dental caries status: A comparative study

**DOI:** 10.6026/973206300221550

**Published:** 2026-03-31

**Authors:** Ashwini Gaikwad, Aniket Jadhav, Rajlaxmi Pati, Abhijit Jadhav, Shivani Chavan

**Affiliations:** 1 Department of Conservative Dentistry and Endodontics, Bharati Vidyapeeth Deemed to be University Dental College & Hospital, Pune, Maharashtra, India

**Keywords:** Buffering capacity, ICDAS, post menopause, salivary flow rate, salivary pH

## Abstract

Some researchers reported decreased salivary flow in post-menopausal women. Therefore, it is of interest to evaluate salivary pH, buffering capacity, salivary flow rate and ICDAS scores in pre-menopausal and post-menopausal women to assess their impact on oral health. Hence, 32 healthy women were included, with 16 pre-menopausal women (mean age 26 years) serving as the control group and 16 post-menopausal women (mean age 52 years) forming the study group. Saliva collection was standardized and salivary flow rate, pH and buffering capacity were measured using established methods, while ICDAS scores were assessed clinically. The results demonstrated a reduction in salivary flow rate and pH in post-menopausal women, along with higher ICDAS scores compared to the control group, although buffering capacity remained within normal limits. Thus, we show that decreased salivary flow in post-menopausal women may contribute to increased dental caries risk.

## Background:

Menopause represents the permanent cessation of menstruation and generally occurs between 45 and 55 years of age [1]. It is associated with decreased estrogen production, leading to systemic manifestations including mucosal changes, xerostomia, osteoporosis and cardiovascular disease [2, 3]. Saliva plays a crucial role in maintaining oral homeostasis and it regulates pH, facilitates remineralization and neutralizes acids produced by plaque bacteria [4]. The buffering action of saliva depends on bicarbonate, phosphate and protein systems [5]. Several studies have evaluated the effect of menopause on salivary parameters and oral health [6]. Some researchers reported decreased salivary flow in post-menopausal women [7, 8], whereas others documented changes in salivary calcium levels and composition [9, 10]. Salivary flow rate, pH and buffering capacity influence caries development by affecting demineralization and remineralization processes therefore, evaluation of salivary alterations in menopausal women is essential [11, 12]. Therefore, it is of interest to compare salivary pH, buffering capacity, salivary flow rate and ICDAS scores in pre-menopausal and post- menopausal.

## Methodology:

This study was conducted on female patients attending the outpatient department of Conservative Dentistry and Endodontics after obtaining approval from the Institutional Ethics Committee. A statistically determined sample of 32 women was included, comprising 16 healthy pre-menopausal women with regular menstruation (mean age 26 years; range 21-31 years) as Group A and 16 healthy post-menopausal women (mean age 52 years) as Group B. Women aged 21-52 years with normal chewing ability and no evidence of xerostomia or salivary gland disorders were included, while individuals with systemic diseases such as diabetes, hypertension, thyroid disorders, those on medications, pan chewers and completely edentulous patients were excluded. Participants completed a questionnaire regarding age, systemic health and menstrual history and informed consent was obtained; they were instructed to refrain from eating, drinking or brushing for at least one hour prior to saliva collection, which was carried out between 9 am and 12 pm. using stimulated saliva obtained by chewing standardized elastic bands. In the control group, saliva was collected during the first three days of menstruation, whereas in the post-menopausal group saliva secreted during the first minute was swallowed and saliva produced over the next five minutes was collected in a graduated test tube. Salivary flow rate was measured in ml/min, salivary pH was determined using a digital pH meter and buffering capacity was assessed by adding 1 ml of saliva to 3 ml of 0.005 M HCl followed by pH measurement and grading as normal (pH 5-7), borderline (pH 4-5) or low (pH <4). ICDAS scores of the mandibular first molars were recorded clinically and the collected data were compiled, tabulated and statistically analyzed using an independent t-test for pH and the Chi-square test for salivary flow rate, buffering capacity and ICDAS scores.

## Results and Discussion:

Menopause is a natural physiological process that typically occurs in women during their fifth decade of life, marking the permanent end of menstruation. As women approach menopause, numerous physiological and hormonal changes occur, primarily due to decreased estrogen production by the ovaries. Estrogen deficiency can lead to a range of issues, including hot flashes, sweating, osteoporosis, cardiovascular disease, cognitive disorders, urogenital infections and changes in skin health [8]. Saliva, a key part of the oral environment, is a dilute watery solution with both inorganic and organic components. It is vital for mastication, swallowing and speech. Within bacterial plaque, where acid production naturally follows bacterial carbohydrate metabolism, saliva aids in regulating pH through various mechanisms [6]. Bicarbonate, phosphate and histidine-rich peptides function as direct buffers within the plaque once they have permeated it. Urea from saliva undergoes conversion by bacterial urease to ammonia, which neutralizes acid. Amino acids and peptides can be decarboxylated to produce monoamines and polyamines, a process that consumes hydrogen ions [8]. Research indicates that saliva from caries-free individuals exhibits higher levels of the flow rate, pH and buffering capacity compared to those prone to caries. Saliva principally influences the caries process, impacting all three components of Keyes' classic Venn diagram of caries etiology the tooth, plaque and substrate. It affects the rate of flow and clearance, pH and buffering capacity, calcium phosphate homeostasis and bacterial metabolism [10]. Key manifestations of saliva's interactions with caries include its adsorption to oral tissues and its role in eliminating substances from the oral cavity. There are number of cross-sectional studies had been done to correlate the compositions and flow of saliva with dental caries [5]. Hence in the present study, saliva was used as a bio-indicator to evaluate the oral health of menopausal women. A neutral to slightly alkaline salivary pH (around 6.7 to 7.0) is optimal for the remineralization process. This pH range helps maintain a balance where the minerals, such as calcium and phosphate, can redeposit into the enamel, strengthening the teeth [5]. The normal buffering capacity of saliva is 3-30 mg/100 ml. It is important for maintaining a constant pH in the mouth and controlling it by neutralizing acids produced by bacteria in the mouth. This neutralization is crucial in preventing enamel erosion. Saliva contains three possible buffer systems: the protein buffer, the phosphate buffer and the carbonic/ bicarbonate buffer [5]. Salivary flow rate is a critical aspect of oral health, as it helps in maintaining the balance of the oral environment, facilitating digestion, protecting teeth from decay and aiding in speech and taste. The Unstimulated (Resting) Flow Rate is the rate at which saliva is produced without any stimulation, such as eating or chewing. Normally, it ranges between 0.3 to 0.4 millilitres per minute (mL/min) [10]. In view of the above, present study was designed to compare and evaluate salivary pH, buffering capacity, salivary flow rate & ICDAS scores in pre-menopausal & post-menopausal women. For this study, total 32 women were selected. Group A (n=16) served as the control group and consisted of 16 healthy women with regular menstruation, with a mean age of 26 years (ranging from 21 to 31). Group B (n=16) included healthy post-menopausal women with a mean age of 52 years. The participants were asked not to consume anything or brush their teeth at least 1 hour before the collection of saliva. A standardized method was followed for saliva collection. Saliva released in the first minute was swallowed and saliva released in the next five minutes was asked to spit into a test tube with gradations. In Group A, sample was collected during first four days of menstruation. Standardised elastic bands were given for chewing which led to the stimulation of saliva which was later on collected in a graduated test tube. The flow rate was measured as ml/min. Digital pH meter was used to determine the salivary ph. Buffering capacity of saliva was measured by adding 1 ml of saliva to 3 ml of 0.005 M of HCl solution followed by measurement of pH with digital pH meter. ICDAS score. Clinical analysis was used to determine the ICDAS score (International Caries index Detection and Assessment System). In order to provide physicians, epidemiologists and researchers with an evidence-based approach that enables standardized caries detection and diagnosis in a variety of settings and circumstances, the International Caries Detection and Assessment approach (ICDAS) was created. The obtained data was statistically evaluated and following inferences were drawn.

Here is a simplified breakdown of the ICDAS scoring system:

[1] Score 0: Sound tooth surface with no evidence of caries.

[2] Score 1: First visual change in enamel, seen only after air drying or restricted to within the confines of a pit or fissure.

[3] Score 2: Distinct visual change in enamel, visible when wet and appearing wider than the fissure/fossa area.

[4] Score 3: Localized enamel breakdown without visible dentin or underlying shadow.

[5] Score 4: Underlying dark shadow from dentin, with or without localized enamel breakdown.

[6] Score 5: Distinct cavity with visible dentin.

[7] Score 6: Extensive distinct cavity with visible dentin.

The system aims to provide a detailed and standardized way of diagnosing and recording caries, helping in both clinical practice and research. Table 1 and 2 (see PDF) shows Comparison of salivary pH, Buffering capacity and Buffering capacity based on salivary pH respectively between Group A (pre-menopausal) and Group B (post-menopausal). The results showed increase in pH in Group B. Low buffering capacity in Group B, with out of 16 samples, 7 samples showed borderline buffering capacity. Also, Buffering capacity based on salivary pH was low in Group B. However, the difference was not statistically significant with any of the parameters. This increase in salivary pH, Low buffering capacity and lower Buffering capacity based on salivary pH in post-menopausal women indicates that the saliva becomes more acidic and also, the capacity to neutralise with buffering capacity is also lowered down. This makes it more prone for caries incidence [5]. The International Caries Detection and Assessment System (ICDAS) is a method used to evaluate and score dental caries (tooth decay) based on their severity [11]. Salivary flow rate was found to be lower in postmenopausal women in the current investigation than in premenopausal women. Mahesh *et al.*'s investigation produced findings that were comparable [8] and additionally, it was shown by Foglio-Bonda *et al.* [9] that post-menopausal women had a considerably lower salivary flow rate than women who were menstruation. Salivary calcium concentration rises after menopause due to increased circulatory release of calcium [6]. Agha-Hosseini *et al.* [11] confirmed the higher concentration of calcium in the saliva of menopausal women, a finding also supported by Sewón *et al.* [10]. An Iranian study on the salivary flow rate and composition of 42 menopausal women, with and without xerostomia (21 cases and 21 controls), revealed that the mean calcium concentration was significantly higher in the cases than in the controls [12]. Various studies have shown that increased levels of ionized calcium in the saliva, along with higher pH, flow rate and poor oral hygiene, elevate the risk of developing periodontitis due to more rapid plaque calcification [13]. Furthermore, teeth of menstruating individuals tend to be more resistant to caries and have a higher number of intact teeth due to the increased remineralization potential of their saliva [14] the elevated salivary calcium levels enhance plaque calcium content, making it more available for remineralization and thus resulting in a lower incidence of caries [15]. A study by Patil *et al.* also stated that decrease in salivary flow rate had an effect on the dental health of post-menopausal women [16]. It is possible to identify menopausal women who have significant postmenopausal symptoms and the gynecologist and dentist can collaborate to treat these women's symptoms [17]. Mishra *et al.* came to the conclusion that postmenopausal women's decreased salivary pH and flow rate causes an increase in OHI-S, DMFT, CPI and LOA [5]. Use of mouth rinse helps prevention of dental caries in these patients [18]. Advancement to knowledge with present study updates that, recent researches underscores a significant, though complex, link between menopause-related estrogen decline and oral health deterioration. Meta-analyses show that postmenopausal women tend to exhibit a higher DMFT (Decayed, Missing and Filled Teeth) index. Advances in preventive dentistry emphasize the importance of regular monitoring of salivary flow and pH, as well as the use of salivary substitutes and fluoride treatments, to better support the oral health of this population.

## Conclusion:

Within the limitations of this study, post-menopausal women exhibited a lower salivary flow rate and higher ICDAS scores suggesting the increased occurrence of early-stage caries in them compared to pre-menopausal women.
